# The Impact of Drug Reimbursement Policy on Rates of Testosterone Replacement Therapy among Older Men

**DOI:** 10.1371/journal.pone.0098003

**Published:** 2014-07-16

**Authors:** Jolanta Piszczek, Muhammad Mamdani, Tony Antoniou, David N. Juurlink, Tara Gomes

**Affiliations:** 1 Leslie Dan Faculty of Pharmacy, University of Toronto, Toronto, Ontario, Canada; 2 Institute for Clinical Evaluative Sciences (ICES), Toronto, Ontario, Canada; 3 Department of Family and Community Medicine, St. Michael’s Hospital, Toronto, Ontario, Canada; 4 Department of Medicine, Division of Clinical Pharmacology and Toxicology, Sunnybrook Health Sciences Centre, Toronto, Ontario, Canada; 5 Institute of Health Policy, Management, and Evaluation, University of Toronto, Toronto, Ontario, Canada; 6 Applied Health Research Center, Li Ka Shing Knowledge Institute of St. Michael’s Hospital, Toronto, Ontario, Canada; University of British Columbia, Canada

## Abstract

**Background:**

Despite a lack of data describing the long-term efficacy and safety of testosterone replacement therapy (TRT), prescribing of testosterone to older men has increased with the availability of topical formulations. The magnitude of this increase and the impact of formulary restrictions on testosterone prescribing are poorly characterized.

**Methods:**

We conducted a time series analysis using the linked health administrative records of men aged 66 years or older in Ontario, Canada between January 1, 1997 and March 31, 2012. We used interventional autoregressive integrated moving average models to examine the impact of a restrictive drug reimbursement policy on testosterone prescribing and examined the demographic profile of men initiating testosterone in the final 2 years of the study period.

**Results:**

A total of 28,477 men were dispensed testosterone over the study period. Overall testosterone prescribing declined 27.9% in the 6 months following the implementation of the restriction policy (9.5 to 6.9 men per 1000 eligible; p<0.01). However, the overall decrease was temporary and testosterone use exceeded pre-policy levels by the end of the study period (11.0 men per 1000 eligible), largely driven by prescriptions for topical testosterone (4.8 men per 1000 eligible). Only 6.3% of men who initiated testosterone had a documented diagnosis of hypogonadism, the main criteria for TRT reimbursement according to the new policy.

**Conclusion:**

Government-imposed restrictions did not influence long-term prescribing of testosterone to older men. By 2012, approximately 1 in every 90 men aged 66 or older was being treated with TRT, most with topical formulations.

## Introduction

Testosterone-replacement therapy (TRT) is increasingly prescribed to older men with non-specific symptoms attributed to age-related declines in circulating testosterone levels. [1], [2] A recent study of younger men with commercial health insurance showed that the rate of TRT use has increased 359% in the United States in the last decade. [3] This trend has occurred despite ongoing ambiguity surrounding the diagnosis of late-onset hypogonadism and the lack of high quality evidence demonstrating the long-term efficacy of TRT. [4]–[7] Specifically, studies examining TRT are limited by short follow-up, [8], [9] small sample sizes [9], [10] and use the of surrogate outcomes such as changes in hormone levels, bone mineral density and measures of muscle strength. [11]–[13] Moreover, the safety of extended TRT is poorly characterized, particularly among older men with multiple comorbidities. [14] Notably, a large randomized trial of TRT among men older community-dwelling men was prematurely discontinued because of a significantly increased risk of cardiovascular events in the treatment group relative to placebo [15] and a recent study of a male Veterans cohort associated TRT use with increased mortality, myocardial infarction and stroke. [16] Regardless, several new testosterone replacement products have been introduced [17] and prescriptions for novel formulations such as topical preparations have also increased rapidly [1].

Numerous regions have attempted to curb testosterone utilization. In Ontario, where older individuals receive universal drug coverage through the provincially funded Ontario Drug Benefits (ODB) program [18], no restrictions were in place governing the use of these drugs prior to the listing of topical TRT in 2005. However, to limit TRT prescribing, in 2006 the provincial government restricted coverage of all formulations of TRT to the treatment of new endocrinopathy occurring at any level of the hypothalamic-pituitary-testicular axis, defined as a “confirmed low morning serum testosterone levels associated with symptomatic testicular disease” [19].

Despite many changes to the availability and diversity of TRT options over the past decade, population-based studies examining the impact of these changes on prescribing trends of these drugs among older men are lacking. We investigated temporal trends in rates of testosterone use among elderly men and the impact of the introduction of prescribing restrictions on the use of these products in Ontario. Finally, we sought to identify the characteristics of men who commenced treatment with TRT to appreciate the presence of comorbidities that could potentially impact the safety of testosterone in this population.

## Methods

### Setting and Design

We conducted a cross-sectional time series analysis examining changes in rates of use of testosterone products reimbursed by the provincial drug plan in Ontario, Canada, from January 1^st^, 1997 to March 31^th^, 2012. Since March 2006, the Ontario Public Drug Plan has reimbursed prescription costs for all testosterone products to men over the age of 65, provided that the prescriber specifies on the prescription that the patient has confirmed low morning serum testosterone levels associated with documented, symptomatic hypothalamic, pituitary or testicular disease, or a diagnosis of HIV. [19] During this time, Ontario had a population of approximately 14 million people; of these, approximately 650,000 were men aged 65 or older with universal access to prescription-drug coverage, physician services and hospital care. [18] This study was approved by the Research Ethics Board of Sunnybrook Health Sciences Centre, Toronto, Ontario.

### Sources of Data

We identified computerized prescription records using the Ontario Drug Benefit database, which identifies prescription drugs dispensed to all Ontario residents 65 years or older. We obtained data pertaining to comorbidities and health resource utilization using hospital admissions data from the Canadian Institute for Health Information (CIHI) Discharge Abstract Database, emergency department visit data from the CIHI National Ambulatory Care Reporting System, and physician billing claims from the Ontario Health Insurance Plan database. We ascertained diabetes diagnoses using the Ontario Diabetes Database and basic demographic information from the Registered Persons Database, a registry of all Ontario residents eligible for health services. We determined the practice specialty of physicians using the Institute for Clinical Evaluative Sciences Physician Database. We used the Institute for Clinical and Evaluative Sciences (ICES) data repository for all sources of data. All databases were linked and analyzed in an anonymous fashion using encrypted patient 10-digit health card numbers and encrypted physician identifiers, and are routinely used to study trends in medication use [20], [21].

### Identification of Patients

We identified men aged 66 years or older who received at least one prescription for a testosterone product over the study period. We excluded the first year of individual eligibility for prescription drug coverage (age 65) to avoid incomplete medication records.

We conducted a time series analysis of prevalent testosterone users in quarterly intervals over the study period. Each quarter, we defined prevalent users as those who filled a prescription for testosterone products, overall and stratified by formulation (oral, topical and injected). All estimates were calculated as rates, adjusted per 1000 eligible elderly, defined as all men over the age of 66 residing in Ontario.

In a secondary analysis, we described the baseline characteristics of patients initiating testosterone therapy in the 2 final years of the study period (April 1^st^, 2010 to March 31^st^, 2012). We focused on new users to avoid comparing men at different stages of illness, defining new users as those who had not received any other prescription for a testosterone product in the previous 365 days. Patient characteristics examined included general demographic information (age, location of residence, income) and cardiovascular conditions (hypertension, heart failure, acute coronary syndrome, stroke) in the previous 3 years, any past diabetes diagnosis, and the specialty of the physician who initiated the testosterone therapy. Diagnoses of hypogonadism in the prior 3 years were identified using physician billing claims (diagnosis code 257) and hospitalization records (10^th^ revision of the International Statistical Classification of Diseases and Related Health Problems (ICD-10) codes E29.1, E89.5 and N52.9). We also examined the Charlson Comorbidity Index and the total number of distinct drugs dispensed in the previous 6 months as two measures to assess patient comorbidity [22], as well as the total number of inpatient hospital, emergency department and physician office visits in the preceding 3 years. Finally, we identified prescriptions for anti-hypertensive agents, statins, oral anti-diabetic agents, insulin, and oral anticoagulants in the previous 6 months.

### Statistical Analysis

In the primary analysis, we conducted time series analysis using autoregressive integrated moving-average (ARIMA) models [23] to examine the impact of the restriction of public drug coverage of testosterone products in March 2006 on the quarterly prevalence of testosterone therapy. We stratified all analyses by testosterone formulation (injectable, oral or topical, the latter including gel and transdermal patches). Seasonality was assessed and taken into account when developing the ARIMA models.

We used the correlograms depicting autocorrelation, partial autocorrelation, and inverse autocorrelation functions to guide initial model selection. We assessed autocorrelation at various lags using the Ljung-Box Chi-square statistic [24] and stationarity using the augmented Dickey-Fuller test. [25] The change in reimbursement status in Q1 2006 was reflected as a step function in the regression model.

In a secondary analysis, we compared baseline characteristics among patients newly initiating oral, topical or injectable testosterone formulations using the one-way ANOVA test for means, Kruskal-Wallis test for medians, and chi-squared test for categorical and binary variables. A p-value<0.05 was considered statistically significant. All analyses were conducted using the SAS software (version 9.2).

## Results

Over the 183 -month study period, 28,477 men aged 66 or older were treated with testosterone therapy. During this time, we identified 292,307 prescriptions for all formulations of TRT prescribed to elderly men reimbursed by the government plan.

### Rates of Testosterone Use

**Figure 1:**
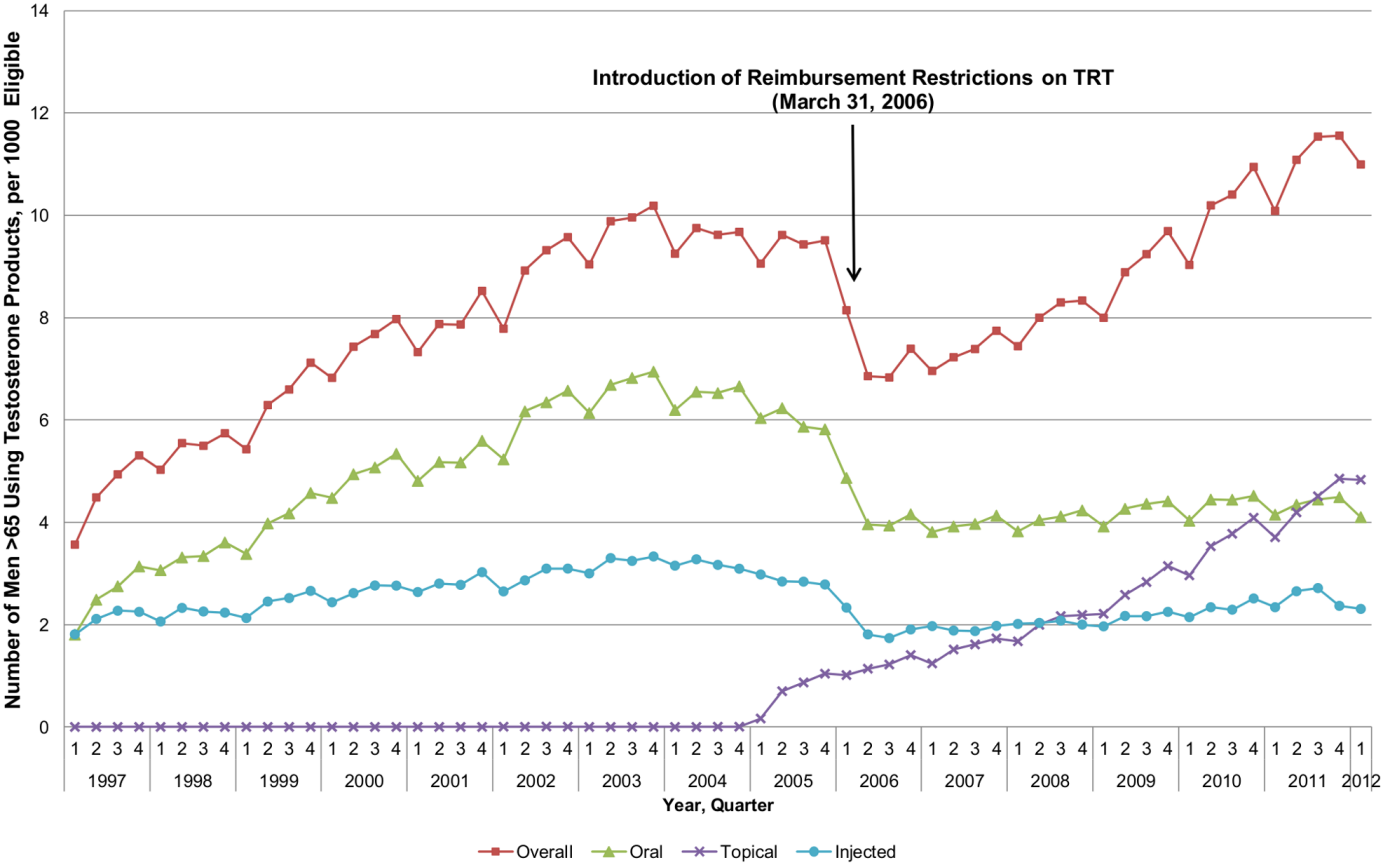
Rate of Testosterone Use per 1,000 Men Eligible for Public Drug Coverage and Aged 65 and older. Figure 1 depicts a steady 286% increase in testosterone use between 1997 and 2003, and a subsequent plateau in 2004 and 2005. In early 2006, the introduction of universal prescribing restrictions for TRT led to a 27.9% drop in total user rates within a 6 month period, driven by the decline of oral (p<0.01) and injectable (p<0.01), but not topical testosterone use. However, after this initial drop, total TRT use started to increase again and by the end of the study period (March 2012) TRT rates had reached a historical peak of 11.0 men per 1000 eligible population. This increase was largely driven by the use of topical testosterone products, while rates of oral and injectable use each fell 32% and 35% respectively after the universal restriction policy was implemented and remained at this new low.

Rates of testosterone use increased steadily, rising 286% between 1997 and 2003 (from 3.6 to 10.2 men per 1000 eligible population), after which they reached a plateau of approximately 9.5 men per 1000 eligible population in 2004 and 2005 (Fig. 1). Prior to 2005, only oral and injectable formulations were available on the provincial formulary, and after a period of the initial growth, user rates of these products stabilized and remained unchanged from early 2004 to the end of 2005 (3.0 men per 1000 eligible population for injectable TRT and 6.2 men per 1000 eligible population for oral TRT). Topical testosterone was added to the provincial formulary in January 2005.

In early 2006, the introduction of universal prescribing restrictions for TRT led to a 27.9% drop in total user rates within a 6 month period (9.5 to 6.9 men per 1000 eligible population), driven by the decline of oral (p<0.01) and injectable (p<0.01), but not topical testosterone use (Fig. 1), which was unaffected. However, following the initial drop, total TRT user rates quickly increased again. By the end of the study period (March 2012) TRT rates had reached a historical peak of 11.0 men per 1000 eligible population, reflecting a 310% increase from the beginning of the study period. This increase was largely driven by the use of topical testosterone products, which rose 464% between the policy change in 2006 and the end of the study period (from 1.0 to 4.8 men per 1000 eligible), while rates of oral and injectable use each fell 32% and 35% respectively and remained at this new low over the same period. By the first quarter of 2012, rates of use of topical TRT (4.8 men per 1000 eligible population) exceeded those of oral and injectable formulations (4.1 and 2.3 men per 1000 population, respectively).

### Baseline Characteristics

We identified 3,718 men aged 66 years or older who initiated TRT between April 1, 2010 and March 31 2012. Among these men, 20.9% (N = 776) initiated injectable, 28.2% (N = 1049) initiated oral and 50.9% (N = 1893) initiated topical TRT formulations. Overall, new TRT users were similar regardless of the type of TRT prescribed (Table 1). Men using TRT had a multitude of comorbidities; 61.2% (N = 2276) had a documented diagnosis of hypertension and 36.0% (N = 1337) had a diagnosis of diabetes. These men were prescribed an average of 9.3 prescription medications in the preceding year, and a large proportion received lipid-lowering (N = 2093, 56.3%) and anti-hypertensive (N = 2496, 67.1%) drugs.

**Table 1:** Baseline Characteristics of Men Newly Prescribed Testosterone between April 1 2010 and March 31 2012.

	Overall	Topical	Injected	Oral
	N = 3,718	N = 1,893	N = 776	N = 1,049
**Age (Mean, SD)**	70.7±5.6	71.1±5.6	70.1±5.5*	70.3±5.4**
**Urban residence**	3,183 (85.6%)	1,631 (86.2%)	664 (85.6%)	888 (84.7%)
**Resident of Long Term Care Facility**	10 (0.3%)	< = 5‡	< = 5‡	< = 5‡
**Income Quintile**				
*1*	587 (15.8%)	274 (14.5%)	132 (17.0%)	181 (17.3%)**
*2*	672 (18.1%)	336 (17.7%)	145 (18.7%)	191 (18.2%)
*3*	685 (18.4%)	344 (18.2%)	137 (17.7%)	204 (19.4%)
*4*	812 (21.8%)	424 (22.4%)	165 (21.3%)	223 (21.3%)
*5*	949 (25.5%)	507 (26.8%)	194 (25.0%)	248 (23.6%)
*Missing*	13 (0.3%)	8 (0.4%)	< = 5‡	< = 5‡
**Number hospitalizations in past** **3 yrs (Mean, SD)**	0.51±1.09	0.53±1.17	0.49±1.03	0.46±0.97
*0*	2,652 (71.3%)	1,333 (70.4%)	561 (72.3%)	758 (72.3%)
*1*	661 (17.8%)	343 (18.1%)	133 (17.1%)	185 (17.6%)
*2 to 4*	354 (9.5%)	188 (9.9%)	71 (9.1%)	95 (9.1%)
*5+*	51 (1.4%)	29 (1.5%)	11 (1.4%)	11 (1.0%)
**Number of Emergency Department** **Visits in past 3 yrs (Mean, SD)**	1.74±3.30	1.76±3.26	1.64±2.60	1.77±3.79
*0*	1,643 (44.2%)	808 (42.7%)	349 (45.0%)	486 (46.3%)
*1*	800 (21.5%)	422 (22.3%)	170 (21.9%)	208 (19.8%)
*2 to 4*	894 (24.0%)	458 (24.2%)	184 (23.7%)	252 (24.0%)
*5+*	381 (10.2%)	205 (10.8%)	73 (9.4%)	103 (9.8%)
**Number of Physician Visits in** **past 3 yrs (Median, IQR)**	31 (20–48)	31 (20–47)	37 (23–56)*	30 (18–43)
**Charlson Score**				
*No Hospitalization*	2,654 (71.4%)	1,334 (70.5%)	562 (72.4%)	758 (72.3%)
*0*	498 (13.4%)	263 (13.9%)	107 (13.8%)	128 (12.2%)
*1*	214 (5.8%)	112 (5.9%)	40 (5.2%)	62 (5.9%)
*2+*	352 (9.5%)	184 (9.7%)	67 (8.6%)	101 (9.6%)
**Comorbidities in past 3 yrs**				
*Congestive Heart Failure*	72 (1.9%)	38 (2.0%)	18 (2.3%)	16 (1.5%)
*Acute Myocardial Infarction*	73 (2.0%)	40 (2.1%)	11 (1.4%)	22 (2.1%)
*Stroke*	27 (0.7%)	15 (0.8%)	9 (1.2%)	< = 5
*Hypertension*	2,276 (61.2%)	1,171 (61.9%)	483 (62.2%)	622 (59.3%)
*Diabetes*	1,337 (36.0%)	677 (35.8%)	291 (37.5%)	369 (35.2%)
*Hypogonadism*	233 (6.3%)	118 (6.2%)	79 (10.2%)*	36 (3.4%)**
**Number of drugs in past 1 yr**	9.33±6.14	9.28±6.09	9.70±6.27	9.14±6.12
**Specialty of Physician Initiating** **Testosterone**				
*General Practitioner*	2,472 (66.5%)	1,138 (60.1%)	568 (73.2%)*	766 (73.0%)**
*Endocrinology*	181 (4.9%)	114 (6.0%)	47 (6.1%)	20 (1.9%)**
*Urology*	573 (15.4%)	364 (19.2%)	51 (6.6%)*	158 (15.1%)**
*Other Specialty*	154 (4.1%)	74 (3.9%)	44 (5.7%)*	36 (3.4%)
*Unknown Specialty*	338 (9.1%)	203 (10.7%)	66 (8.5%)	69 (6.6%)**
**Drug Use in past 6 months**				
*Any Hypertensive*	2,496 (67.1%)	1,305 (68.9%)	505 (65.1%)	686 (65.4%)
*Angiotensin Converting Enzyme Inhibitors*	1,247 (33.5%)	635 (33.5%)	268 (34.5%)	344 (32.8%)
*Angiotensin Receptor Blockers*	837 (22.5%)	448 (23.7%)	149 (19.2%)*	240 (22.9%)
*Beta Blockers*	899 (24.2%)	488 (25.8%)	190 (24.5%)	221 (21.1%)**
*Calcium Channel Blockers*	893 (24.0%)	474 (25.0%)	190 (24.5%)	229 (21.8%)
*Diuretics*	1,187 (31.9%)	612 (32.3%)	242 (31.2%)	333 (31.7%)
*Statins*	2,093 (56.3%)	1,096 (57.9%)	430 (55.4%)	567 (54.1%)**
*Oral hypoglycemic agents*	831 (22.4%)	423 (22.3%)	171 (22.0%)	237 (22.6%)
*Insulin*	243 (6.5%)	126 (6.7%)	60 (7.7%)	57 (5.4%)
*Anticoagulants*	303 (8.1%)	161 (8.5%)	64 (8.2%)	78 (7.4%)

*Indicates a statistically significant difference (p-value<0.05) when comparing injectable to topical patient group.

**Indicates a statistically significant difference (p-value<0.05) when comparing oral to topical patient group.

‡ Cells suppressed due to small numbers to protect the privacy of patients.

Among men who commenced testosterone therapy, only 6.3% (N = 233) had a recorded diagnosis of hypogonadism in the past 3 years. However, this varied by testosterone formulation; patients initiated on injectable formulations of testosterone were significantly more likely to have this diagnosis (10.2%, relative to topical (6.2%) and oral (3.4%) testosterone users (p<0.05). Further, although family physicians initiated TRT for most men (n = 2,473; 66.5%), urologists were the most likely to chose topical testosterone for new users (N = 364, 19.2%), compared with injectable (N = 158, 15.1%; p<0.05) and oral formulations (N = 51, 6.6%; p<0.05). Overall, endocrinologists prescribed testosterone therapy to only 4.9% (N = 181) of men newly initiated on TRT.

## Interpretation

In this study spanning 15 years, we demonstrated a substantial increase in the use of TRT over time, despite the lack of long-term efficacy and safety data in elderly men. Acknowledging this uncertainty and restricting the criteria for TRT reimbursement on the public drug formulary led to a sharp decline in use; however this decline was temporary as TRT utilization resumed its upward trend following the introduction of topical TRT. By 2010, the rate of testosterone use exceeded the earlier peak rate and continued to rise thereafter. By the first quarter of 2012, one in every 90 men aged 66 and older were being treated with testosterone. This finding indicates that this policy, although designed to restrict inappropriate prescribing of TRT, was only briefly effective by initially lowering rates of injectable and oral testosterone use, and relatively ineffective in curtailing growth of topical testosterone products. This contrasts with a previous analysis of a reimbursement restriction policy in Ontario, which has been successful in limiting inappropriate prescribing of fluoroquinolones over a longer term [26], but is consistent with an Australian analysis of oral and injectable TRT prescription rates, which showed that introducing a mandatory phone call to authorize a TRT prescription resulted in only a partial and temporary curtailment. [27] Finally, our study shows that TRT, which carries potential safety concerns, is being prescribed to elderly men with a variety of comorbidities who differ substantially from younger, healthier men generally included in clinical trials of these products. [7]–[10] Of particular concern is the large proportion of TRT users being treated for cardiovascular risk factors such as hypertension, diabetes and dyslipidemia considering the new evidence of the potential association between testosterone replacement and myocardial infarction and stroke [15], [16].

Several factors may have contributed to the increasing use of TRT observed in this study and the lack of sustained effect of the drug reimbursement policy. First, TRT has been aggressively marketed to both clinicians and patients for non-approved indications such as ‘andropause’ and male sexual dysfunction. [28]–[31] In fact, in the United States, where direct to consumer advertising is legal, the rates of TRT use are three times higher than those observed in our study. [3] This is consistent with our observation that a low proportion of men had a documented diagnosis of testicular dysfunction. In addition, topical TRT is more convenient [32] and produces more stable drug levels than oral or injectable formulations [33], properties that may increase patient and clinician acceptance of testosterone. In addition, a TRT manufacturer recently published a study urging clinicians to improve adherence to topical testosterone, promoting long-term treatment for unofficial conditions such as age-related androgen decline. [34] Indeed, in our study we found that rates of topical TRT use have increased consistently since its introduction in 2005 as compared to rates of oral and injectable TRT use, which have remained relatively stable since 2006. Finally, publications related to testosterone use are given considerable exposure in the lay and medical press, which further drives medicalization of aging, drug campaigns and development of new products [35], [36].

Our study has some limitations that merit emphasis. First, our data do not include patients younger than 65 years of age who may also use TRT and so may not be generalizable to the entire population of men in Ontario. A recent study of IMS data by Handelsman et al. found high volumes of TRT sales in Canada that exceed the prescription rates reported in our study. [37] It is likely that this discrepancy is driven by the different study populations in these two studies. Handelsman et al. included all prescriptions sales for TRT in Canada, which capture prescriptions dispensed to younger men, as well as those paid for through private drug plans and cash payments. However, the authors not distinguish between testosterone sold to be used in Canada and the large proportions of sales from internet pharmacies intended for export. As a result, it is unsurprising that we observed lower rates of use in our study, as prescribing was restricted to older men in a public drug program where some prescribing restrictions (albeit unenforced) are place. [37] Second, although the government plan notionally reimburses TRT only for men who meet pre-specified diagnostic criteria, no objective serum testosterone level or formal application process is required and physicians are not subject to review or auditing processes. Due to this, and our lack of access to laboratory data that precluded verification of patients’ serum testosterone levels, the extent to which prescribers comply with these restrictions is unknown. Indeed, the results of this study suggest that TRT is frequently being prescribed to patients who do not meet these criteria. Third, to predict TRT usage rates, our model was fixed around the event of introducing prescribing restrictions. It is possible that other factors unbeknownst to us could have contributed to the decline of TRT use at the time of this policy change. Finally to identify men with hypogonadism, we used OHIP and ICD-10 billing codes for testicular dysfunction which have not been validated, and may underestimate or overestimate the number of men with this diagnosis.

In conclusion, treatment with testosterone is re-gaining popularity despite attempts to restrict prescribing, and topical testosterone has quickly become the most popular formulation of TRT used among older men. This is particularly concerning because these men carry a significant burden of illness, and there is a paucity of data regarding the long-term efficacy and safety of this drug among men with multiple comorbidities and high medication use.
